# Rap1a Activity Elevated the Impact of Endogenous AGEs in Diabetic Collagen to Stimulate Increased Myofibroblast Transition and Oxidative Stress

**DOI:** 10.3390/ijms23094480

**Published:** 2022-04-19

**Authors:** Stephanie D. Burr, Christopher C. Dorroh, James A. Stewart

**Affiliations:** Deptrtment of BioMolecular Sciences, School of Pharmacy, University of Mississippi, Oxford, MS 38677, USA; ccdorroh@go.olemiss.edu (C.C.D.); jastewa7@olemiss.edu (J.A.S.J.)

**Keywords:** Rap1a, AGE/RAGE signaling, hyperglycemia, diabetes, cardiac fibroblasts, myofibroblasts, oxidative stress, extracellular matrix

## Abstract

Diabetics have an increased risk for heart failure due to cardiac fibroblast functional changes occurring as a result of AGE/RAGE signaling. Advanced glycation end products (AGEs) levels are higher in diabetics and stimulate elevated RAGE (receptor for AGE) signaling. AGE/RAGE signaling can alter the expression of proteins linked to extracellular matrix (ECM) remodeling and oxidative stressors. Our lab has identified a small GTPase, Rap1a, that may overlap the AGE/RAGE signaling pathway. We sought to determine the role Rap1a plays in mediating AGE/RAGE changes and to assess the impact of isolated collagen on further altering these changes. Primary cardiac fibroblasts from non-diabetic and diabetic mice with and without RAGE expression and from mice lacking Rap1a were cultured on tail collagen extracted from non-diabetic or diabetic mice, and in addition, cells were treated with Rap1a activator, EPAC. Protein analyses were performed for changes in RAGE-associated signaling proteins (RAGE, PKC-ζ, ERK1/2) and downstream RAGE signaling outcomes (α-SMA, NF-κB, SOD-2). Increased levels of endogenous AGEs within the diabetic collagen and increased Rap1a activity promoted myofibroblast transition and oxidative stress, suggesting Rap1a activity elevated the impact of AGEs in the diabetic ECM to stimulate myofibroblast transition and oxidative stress.

## 1. Introduction

Heart failure can arise in diabetics when cardiac fibroblasts transition to “activated” fibroblasts (i.e., myofibroblasts) to increase myocardial extracellular matrix (ECM) remodeling contributing to left ventricular (LV) hypertrophy [1,2,3]. Myofibroblasts are characterized by increased alpha smooth muscle actin (α-SMA) protein expression [4,5,6,7]. Increased myofibroblast populations under diabetic conditions also promote signaling cascades to increase reactive oxygen species (ROS), inflammatory proteins, and ECM remodeling [8,9,10,11,12]. Of the key signaling cascades contributing to these events, the AGE/RAGE signal cascade, where AGEs (advanced glycated end products) accumulate in high glucose levels and activate their receptor (RAGE, receptor for advanced glycated end products), to elicit intracellular signaling [13,14,15]. AGEs form crosslinks between collagen fibers and elicit RAGE activation, leading to a complex signaling network with multiple downstream end points [12,16,17,18]. Furthermore, studies have shown that collagen isolated from diabetic mice had significantly more AGEs than non-diabetic mice and was similar levels of AGEs in diabetic individuals [4,19,20]. 

ROS production is a major outcome of RAGE signaling resulting from activation of protein kinase C-zeta (PKC-ζ) to alter NADPH (nicotinamide adenine dinucleotide phosphate) oxidase activity [21,22,23]. Elevated AGE/RAGE signaling and ROS production increases activity of signaling molecules, such as superoxide induction of ERK1/2 (extracellular signal related-kinase 1/2) activity and subsequently activated nuclear factor kappa-light-chain-enhancer of activated B cells (NF-κB) perpetuating changes in oxidative stress and inflammation [24,25,26,27,28,29]. NF-κB, PKC-ζ, and ERK1/2 alter superoxide dismutases (SODs) to decrease ROS concentrations [17,26,30,31,32]. Li et al., 2006 showed mesangial cells exposed to AGEs stimulated ROS production to intensify ECM remodeling via ERK activation, and treatment with SODs reduced ROS effects [12]. In our lab, we have identified a small GTPase, Rap1a, that appears to overlap and regulate expression and activity of AGE/RAGE signaling proteins [20,33].

Rap1a (repressor/activator protein 1A) is a small GTPase of the Ras superfamily connecting extracellular signals to intracellular responses [34,35,36]. Rap1a activity can be triggered by exchanged protein activated by cyclic AMP (EPAC) [37]. In adult myocardium, Rap1a can induce changes in cellular function, such as PKC-ERK-mediated migration [38,39]. Rap1a, ERK1/2, and PKC are significant members of the AGE/RAGE signaling cascade and its downstream events altering ECM remodeling, inflammation, and oxidative stress [25,33,40,41]. 

The ECM is a major contributor to cellular physiology acting as physical support and reservoir for signaling factors affecting cellular behavior and function [42,43]. Binding of integrin α1β1 to type I collagen in isolated microvascular endothelial cells decreased cAMP and PKA (protein kinase A) expression leading to increased F-actin stress fibers [44]. While the impact of ECM components on cellular responses have been investigated, few studies have examined the role of ECM isolated from an in vivo diabetic source on cell behavior, phenotype changes, and fibroblast function. 

This study aims to better understand the effects of isolated ECM components and endogenous AGE exposure on RAGE-associated signaling proteins in cardiac fibroblasts. Specifically, we sought to (1) examine the impact of the diabetic, endogenous AGE-crosslinked ECM on RAGE signaling and (2) determine the role Rap1a in modulating changes induced by endogenous AGEs within the diabetic ECM via the AGE/RAGE signaling cascade. We hypothesized endogenous AGEs in combination with the diabetic ECM will increase RAGE-associated signaling protein expression, and these changes will be mediated by Rap1a. To accomplish this goal, we isolated cardiac fibroblasts from genetically different mice and cultured them on collagen isolated from non-diabetic or diabetic mice. Cells were treated with EPAC to stimulate Rap1a signaling mechanisms. Alterations in expression of proteins linked to RAGE associated signaling outcomes were assessed. These results determined that endogenous AGEs within the ECM induced changes in protein expression in cardiac fibroblasts, and these effects were mediated by Rap1a altering the AGE/RAGE signaling cascade.

## 2. Results

### 2.1. Diabetic Cardiac Fibroblasts Exhibited Higher Levels of RAGE-Associated Signaling Proteins Compared to Non-Diabetic and Rap1a Fibroblasts

**Figure 1:**
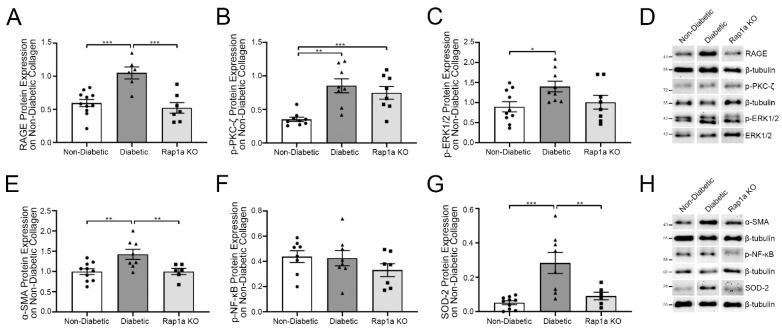
Diabetic cardiac fibroblasts exhibited higher levels of RAGE-associated signaling proteins compared to non-diabetic and Rap1a fibroblasts. Non-diabetic, diabetic, and Rap1a KO cardiac fibroblasts were isolated and P1 fibroblasts were cultured on collagen isolated from non-diabetic mouse tails. Total protein was collected from fibroblasts and assessed for changes in protein expression between non-diabetic, diabetic, and Rap1a KO fibroblasts. RAGE signaling proteins were determined by analyzing protein expression of (**A**) (**G**) RAGE (46 kDa), (**B**) p-PKC-ζ (72 kDa), and (**C**) p-ERK1/2 (44 and 42 kDa, respectively). (**D**) Representative Western blot images for RAGE, p-PKC-ζ, and p-ERK1/2, are presented. Downstream targets of AGE/RAGE signaling were assessed by examining protein expression for (**E**) α-SMA (42 kDa), (**F**) p-NF-κB (65 kDa), and (**G**) SOD-2 (25 kDa). (**H**) Representative Western blot images for α-SMA, p-NF-κB, and SOD-2, and all Western blot images are not displayed as continuous blots due to logistical constraints brought on by the number of treatment groups and genotypes. The original Western blot images are available at doi:10.6084/m9.figshare.13369535. Protein expression for α-SMA, p-NF-κB, SOD-2, p-PKC-ζ, and RAGE was normalized to β-tubulin (55 kDa), and p-ERK1/2 protein expression was normalized to total ERK1/2 protein expression. Mean ± SEM are depicted on graph (*n* = 6–11) and significance was determined by one-way ANOVA followed by a Fisher’s Protected Least Significant Difference post hoc (* *p* < 0.05, ** *p* < 0.01, and *** *p* < 0.001).

Previous studies by our laboratory have established that collagen isolated from mouse tails contained endogenous AGEs [4,20]. Based on these findings, we examined the impact of non-diabetic collagen on non-diabetic, diabetic, and Rap1a KO cardiac fibroblasts by evaluating changes in the expression of RAGE cascade-associated signaling proteins (Figure 1). RAGE protein expression was significantly higher in diabetic fibroblasts compared to both non-diabetic and Rap1a KO cells (Figure 1A; one-way ANOVA *p* = 0.0002). RAGE signaling protein, p-PKC-ζ, was significantly higher in diabetic cells compared to non-diabetic fibroblasts but not in Rap1a KO cells (Figure 1B; one-way ANOVA *p* = 0.0004). The expression of p-PKC-ζ was significantly higher in Rap1a KO cells compared to non-diabetic cells. Diabetic cardiac fibroblasts exhibited significantly more p-ERK1/2 protein expression compared to non-diabetic cells but not Rap1a KO cells (Figure 1C; one-way ANOVA *p* = 0.0444). Diabetic fibroblasts displayed significantly more α-SMA protein expression than non-diabetic and Rap1a KO fibroblasts (Figure 1E; one-way ANOVA *p* = 0.0053). In comparison, p-NF-κB expression was not significantly greater in diabetic fibroblasts compared to non-diabetic and Rap1a KO cells (Figure 1F; one-way ANOVA *p* = 0.1095). Oxidative stress indicator, SOD-2, was significantly higher in diabetic fibroblasts compared to non-diabetic and Rap1a KO cells (Figure 1G; one-way ANOVA *p* = 0.0004). These results showed that diabetic cardiac fibroblasts have elevated expression of AGE/RAGE-associated proteins compared to non-diabetic and Rap1a KO cells.

### 2.2. On Non-Diabetic Collagen, RAGE-Associated Cascade Signal Proteins in Diabetic RKO Fibroblasts Did Not Differ from Non-Diabetic RKO Fibroblasts

**Figure 2:**
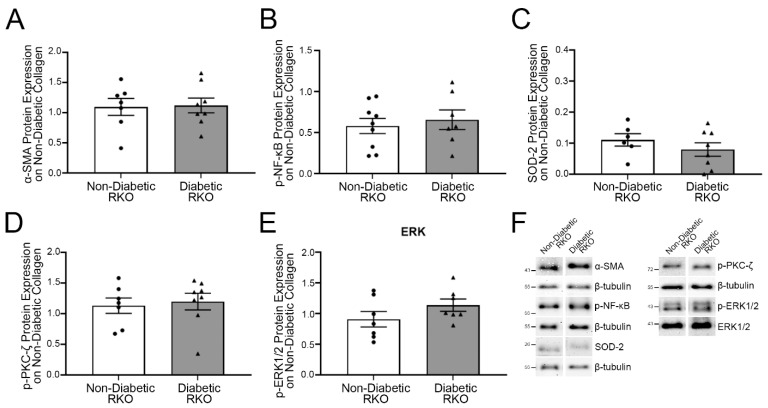
On non-diabetic collagen, RAGE-associated cascade signal proteins in diabetic RKO fibroblasts did not differ from non-diabetic RKO fibroblasts. Cardiac fibroblasts (P1) isolated from non-diabetic and diabetic RKO mice were cultured on collagen isolated from non-diabetic mice. Total protein was collected and used to evaluate the expression of AGE/RAGE pathway proteins involved with the signaling cascade and downstream outcomes: (**A**) α-SMA (42 kDa), (**B**) p-NF-κB (65 kDa), (**C**) SOD-2 (25 kDa), (**D**) p-PKC-ζ (72 kDa), and (**E**) p-ERK1/2 (44 and 42 kDa, respectively). (**F**) Representative Western blot images for α-SMA, p-NF-κB, SOD-2, p-PKC-ζ, and p-ERK1/2 are shown and are not displayed as continuous blots due to logistical constraints brought on by the number of treatment groups and genotypes. The original Western blot images are available at doi:10.6084/m9.figshare.13369535. Protein expression for α-SMA, p-NF-κB, SOD-2, and p-PKC-ζ was normalized to β-tubulin (55 kDa) and p-ERK1/2 was normalized to total ERK1/2 protein expression. Mean ± SEM are presented in the graphs (*n* = 6–9) and statistical differences were determined using a Student’s *t*-test.

In order to determine if changes noted previously were due to the presence of collagen or the AGEs within the collagen, protein expression was examined in RAGE knockout (RKO) cardiac fibroblasts (Figure 2). Diabetic RKO fibroblasts did not have significantly more α-SMA expression compared to non-diabetic RKO cells when plated on non-diabetic collagen (Figure 2A; Student’s *t*-test, *p* = 0.8942). Similarly, expression of both p-NF-κB and SOD-2 was not significantly greater in diabetic RKO cells compared to non-diabetic RKO fibroblasts (Figure 2B,C; Student’s *t*-test, *p* = 0.6171 and *p* = 0.3322, respectively). Lastly, the expression of p-PKC-ζ and p-ERK1/2 did not differ between non-diabetic and diabetic RKO fibroblasts (Figure 2D,E; Student’s *t*-test, *p* = 0.7299 and *p* = 0.8376, respectively). These data indicated AGEs within the collagen were responsible for changes in protein expression.

### 2.3. Exposing Fibroblasts to AGEs in Diabetic Collagen and EPAC Increased Expression of RAGE Associated Signaling Proteins

**Figure 3:**
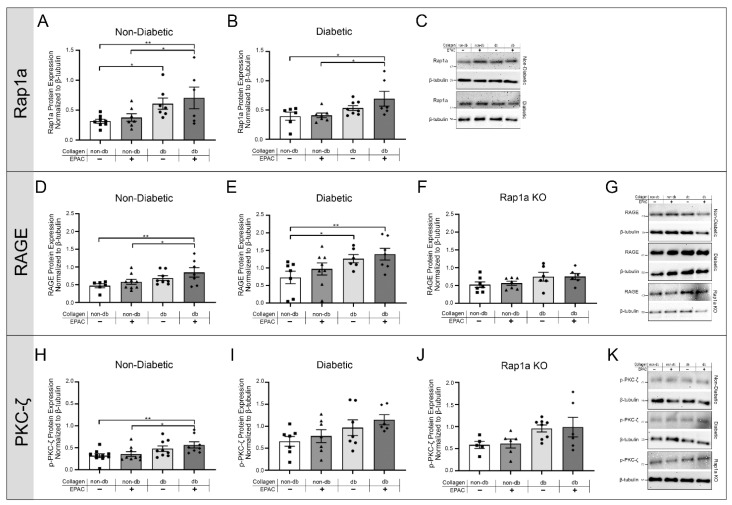
Exposing fibroblasts to AGEs in diabetic collagen and EPAC increased expression of RAGE-associated signaling proteins. Cardiac fibroblasts (P1) isolated from non-diabetic, diabetic, and Rap1a KO mouse hearts were cultured on either non-diabetic or diabetic collagen. Fibroblasts were either left untreated or treated with EPAC for 24 h (100 µM). Total protein was used to assess changes in AGE/RAGE-associated proteins. (**A**–**C**) Rap1a (21 kDa), (**D**–**G**) RAGE (46 kDa), and (**H**–**K**) p-PKC-ζ (72 kDa) protein expression was determined in non-diabetic, diabetic, and Rap1a KO fibroblasts. Protein expression was normalized to β-tubulin (55 kDa). Mean ± SEM was depicted on the graphs (*n* = 6–10). (**C**,**G**,**K**) Representative Western blot images for Rap1a, RAGE, and p-PKC-ζ are depicted. Statistical analysis consisted of one-way ANOVA followed by a Fisher’s Protected Least Significant Difference post hoc (* *p* < 0.05 and ** *p* < 0.01).

Previous studies by our laboratory have established diabetic collagen as having significantly higher levels of AGEs compared to non-diabetic collagen [4,20]. Due to differences in AGE expression, diabetic collagen was used to determine if RAGE activation by endogenous collagenous AGEs could induce changes in protein expression of RAGE-associated signaling proteins. Non-diabetic, diabetic, and Rap1a KO cardiac fibroblasts were cultured on either non-diabetic or diabetic collagen and were then assessed for differences in the expression of proteins associated with AGE/RAGE signaling (Figure 3). Non-diabetic fibroblasts cultured on diabetic collagen with and without EPAC treatment exhibited significantly more Rap1a expression compared to non-diabetic cells cultured on non-diabetic collagen (Figure 3A; one-way ANOVA *p* = 0.0278). Diabetic cells only displayed significantly higher Rap1a expression when cultured on diabetic collagen with EPAC as compared to diabetic cells cultured on non-diabetic collagen (Figure 3B; one-way ANOVA *p* = 0.0340). Non-diabetic RKO and diabetic RKO fibroblasts were assessed for changes in Rap1a expression under the same treatment conditions; however, western results for RKO fibroblasts indicated extremely low to no amounts of Rap1a protein; the bands were too faint to conduct analysis (doi:10.6084/m9.figshare.13369535).

To determine if changes in Rap1a expression were linked to AGE/RAGE signaling, RAGE expression was examined in cardiac fibroblasts grown on non-diabetic and diabetic collagen with or without EPAC treatment (Figure 3D–F). Non-diabetic cells on diabetic collagen with EPAC treatment displayed significantly more RAGE protein expression compared to non-diabetic fibroblasts cultured on non-diabetic collagen either with or without EPAC treatment (Figure 3F; one-way ANOVA *p* = 0.0432). Diabetic fibroblasts grown on diabetic collagen, with and without EPAC, had significantly more RAGE expression when compared to diabetic cells cultured on non-diabetic collagen without EPAC (Figure 3E; one-way ANOVA *p* = 0.0412). Rap1a KO fibroblasts had no significant changes in RAGE expression when cultured on diabetic collagen as compared to Rap1a KO cells cultured on non-diabetic collagen (Figure 3F; one-way ANOVA *p* = 0.1659). EPAC treatment on Rap1a KO cells also had no impact on RAGE expression.

Due to changes in RAGE expression being found, further investigation into signaling proteins involved in the AGE/RAGE cascade was conducted, specifically the expression of p-PKC-ζ and p-ERK1/2 (Figure 3H–J and Supplementary Figure S2). Culturing non-diabetic fibroblasts on diabetic collagen with EPAC treatment caused significantly more p-PKC-ζ expression in comparison to non-diabetic cells cultured on non-diabetic collagen with and without EPAC treatment (Figure 3H; one-way ANOVA *p* = 0.0196). Diabetic cells and Rap1a KO cells cultured on diabetic collagen with EPAC did not result in significant increases in p-PKC-ζ expression compared to cells cultured on non-diabetic collagen (Figure 3I,J; one-way ANOVA *p* = 0.1147 and *p* = 0.0787, respectively). Non-diabetic RKO and diabetic RKO fibroblasts did not exhibit any changes in p-PKC-ζ expression when cultured on either non-diabetic or diabetic collagen or when treated with EPAC (Supplementary Figure S1; one-way ANOVA *p* = 0.9900 and *p* = 0.9807, respectively). There were no significant changes in p-ERK1/2 expression with exposure to endogenous AGEs with or without treatment of EPAC in non-diabetic, diabetic, Rap1a KO, non-diabetic RKO, and diabetic RKO cardiac fibroblasts (Supplementary Figure S2; one-way ANOVA; *p* = 0.1337, *p* = 0.7183, *p* = 0.8653, *p* = 0.9917, and *p* = 0.8653, respectively). The results suggested that diabetic collagen with EPAC treatment caused the highest level of expression of p-PKC-ζ, a RAGE-associated signaling protein.

### 2.4. Endogenous AGEs and Rap1a Activation Altered α-SMA Protein Expression in Both Non-Diabetic and Diabetic Fibroblasts

**Figure 4:**
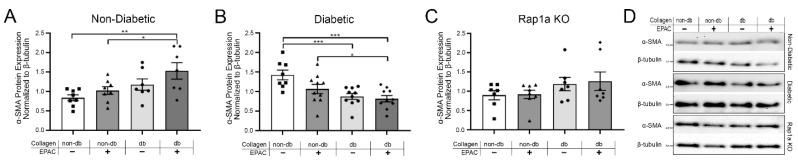
Endogenous AGEs and Rap1a activation altered α-SMA protein expression in both non-diabetic and diabetic fibroblasts. Cardiac fibroblasts were isolated and cultured on non-diabetic or diabetic collagen as well as treated with EPAC (100 µM). Changes in α-SMA (42 kDa) protein expression in (**A**) non-diabetic, (**B**) diabetic, and (**C**) Rap1a KO cardiac fibroblasts were assessed. (**D**) Representative Western blot images for α-SMA expression. α-SMA protein expression was normalized to β-tubulin (55 kDa). Mean ± SEM are presented in graphs with an *n* = 6–10. Statistical analysis consisted of one-way ANOVA followed by a Fisher’s Protected Least Significant Difference post hoc (* *p* < 0.05, ** *p* < 0.01, and *** *p* < 0.001).

As a result of changes to RAGE-associated signaling proteins, we further examined changes in the expression of proteins demonstrated to be upregulated as a result of increased activation of the AGE/RAGE signaling cascade, specifically α-SMA (Figure 4). Non-diabetic fibroblasts exhibited significantly more α-SMA expression when cultured on diabetic collagen and when treated with EPAC as compared to non-diabetic cells cultured on non-diabetic collagen either with or without EPAC (Figure 4A; one-way ANOVA *p* = 0.0114). In contrast, diabetic cells on diabetic collagen had significantly less α-SMA expression compared to diabetic cells on non-diabetic collagen (Figure 4B; one-way ANOVA *p* = 0.0010). Treatment of diabetic cells with EPAC significantly reduced α-SMA expression when cells were cultured on diabetic collagen as compared to diabetic cells on non-diabetic matrix with and without EPAC. Cardiac fibroblasts from both non-diabetic and diabetic RKO mice did not exhibit any changes in α-SMA expression when cultured on either non-diabetic or diabetic collagen (Supplementary Figure S3A–C; one-way ANOVA *p* = 0.9758 and *p* = 0.9924, respectively). Rap1a KO fibroblasts on diabetic collagen did not display a significant difference in α-SMA expression when cultured on diabetic collagen compared to non-diabetic collagen (Figure 4C; one-way ANOVA *p* = 0.3161). The results showed non-diabetic and Rap1a KO cells displayed increased α-SMA expression with diabetic collagen, while diabetic cells saw a reduction in α-SMA expression.

### 2.5. Endogenous AGE Exposure and Increased Rap1a Activity Caused Elevated p-NF-κB Protein Expression

**Figure 5:**
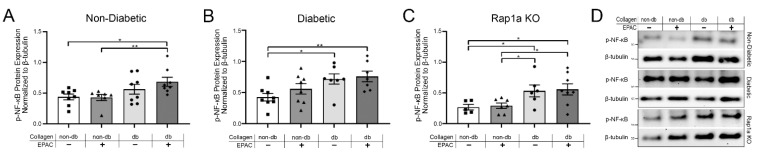
Endogenous AGE exposure and increased Rap1a activity caused elevated p-NF-κB protein expression. (**A**) Non-diabetic, (**B**) diabetic, and (**C**) Rap1a KO cardiac fibroblasts (P1) were cultured on either non-diabetic or diabetic collagen. Fibroblasts were either left untreated or treated with EPAC (100 µM) and total protein was isolated. p-NF-κB (65 kDa) protein expression was assessed and normalized to β-tubulin (55 kDa) protein expression. Data presented in graphs are mean ± SEM (*n* = 6–9). Significant differences were determined using one-way ANOVA followed by a Fisher’s Protected Least Significant Difference post hoc (* *p* < 0.05 and ** *p* < 0.01). (**D**) Representative Western blot images for p-NF-κB expression.

The expression of p-NF-κB was assessed in cardiac fibroblasts cultured on isolated non-diabetic and diabetic collagen. Cardiac fibroblasts with functional RAGE exhibited increased p-NF-κB expression when cultured on diabetic collagen (Figure 5). Non-diabetic fibroblasts showed a significant increase in p-NF-κB expression when cultured on diabetic collagen and treated with EPAC as compared to when non-diabetic cells were cultured on non-diabetic collagen either with or without EPAC treatment (Figure 5A; one-way ANOVA *p* = 0.0309). Further, diabetic fibroblasts exhibited an increase in p-NF-κB expression when cultured on diabetic collagen with and without EPAC as compared to diabetic cells on non-diabetic collagen (Figure 5B; one-way ANOVA *p* = 0.0206). Examination of non-diabetic and diabetic RKO cardiac fibroblasts indicated no change in p-NF-κB expression between the different treatment groups (Supplementary Figure S3D–F; one-way ANOVA *p* = 0.9793 and *p* = 0.9924, respectively). Rap1a KO cells exhibited significantly more p-NF-κB expression when cultured on diabetic collagen, and EPAC treatment did not induce changes in p-NF-κB in Rap1a KO cells (Figure 5C; one-way ANOVA *p* = 0.0230). The data indicated that increased AGEs in diabetic collagen caused an increase in p-NF-κB expression in cells with functional RAGE.

### 2.6. Treatment with EPAC and Diabetic Collagen Caused Decreased SOD Expression in Cardiac Fibroblasts

**Figure 6:**
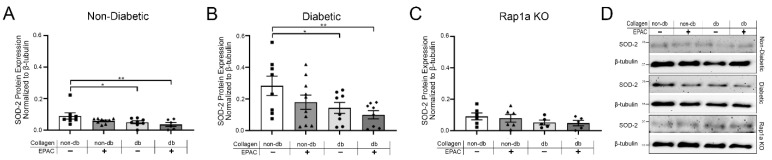
Treatment with EPAC and diabetic collagen caused decreased SOD-2 expression in cardiac fibroblasts. Isolated cardiac fibroblasts from (**A**) non-diabetic, (**B**) diabetic, and (**C**) Rap1a KO were cultured on non-diabetic or diabetic collagen and treated with EPAC (100 µM). Total protein was collected and expression of oxidative maker SOD-2 (25 kDa) was determined. SOD-2 protein expression was normalized to β-tubulin (55 kDa) and mean ± SEM are presented within the graphs (*n* = 5–10). One-way ANOVA followed by a Fisher’s Least Significant Difference post hoc was used to determine significant differences (* *p* < 0.05 and ** *p* < 0.01). (**D**) Representative Western blot images are presented for SOD-2 expression.

The expression of SOD-2 was examined in cardiac fibroblasts cultured on isolated collagen (Figure 6). Non-diabetic cells on diabetic collagen with and without EPAC treatment displayed significantly less SOD-2 expression compared to non-diabetic cells on non-diabetic collagen (Figure 6A; one-way ANOVA *p* = 0.0436). Similarly, diabetic fibroblasts on diabetic collagen had significantly less SOD-2 expression, which was further decreased with EPAC treatment when compared to diabetic cells on non-diabetic collagen (Figure 6B; one-way ANOVA *p* = 0.0482). Non-diabetic RKO and diabetic RKO cells did not display any differences in SOD-2 between treatment groups (Supplementary Figure S3G–I; one-way ANOVA *p* = 0.8768 and *p* = 0.9753, respectively). Further, Rap1a KO cells did not exhibit any significant differences in SOD-2 expression when cultured on diabetic collagen compared to Rap1a KO fibroblasts cultured on non-diabetic collagen (Figure 6C; one-way ANOVA *p* = 0.4242). In addition to SOD-2, SOD-1 expression was assessed in cardiac fibroblasts (Supplementary Figure S4). Cardiac fibroblasts with RAGE showed a decreasing trend in SOD-1 expression when cultured on diabetic collagen but only diabetic cells were significant (Supplementary Figure S4A–C; one-way ANOVA non-diabetic *p* = 0.1823, diabetic *p* = 0.0172, and Rap1a KO *p* = 4298). RKO fibroblasts showed no change in SOD-1 expression between the different treatments (Supplementary Figure S4E,F; one-way ANOVA non-diabetic RKO *p* = 0.9740 and diabetic RKO *p* = 0.9414). These results suggest that increased levels of AGEs in diabetic collagen caused a decrease in the expression of SODs in cells with functional RAGE.

### 2.7. Endogenous AGEs and EPAC Treatment Caused a Decrease in Hydrogen Peroxide in Cardiac Fibroblast with Functional RAGE and Rap1a

**Figure 7:**
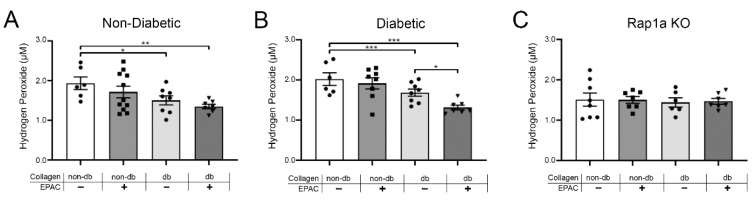
Endogenous AGEs and EPAC treatment caused a decrease in hydrogen peroxide in cardiac fibroblast with functional RAGE and Rap1a. (**A**) Non-diabetic, (**B**) diabetic, and (**C**) Rap1a KO cardiac fibroblasts (P1) were cultured on either non-diabetic or diabetic collagen. Fibroblasts were treated with EPAC (100 µM) for 24 h, followed by isolation of protein lysates. Hydrogen peroxide concentration (µM) was determined for each sample and mean ± SEM were depicted in the graphs (*n* = 6–11). Statistical significance was determined by a one-way ANOVA followed by a Fisher’s Protected Least Significant Difference post hoc (* *p* < 0.05, ** *p* < 0.01, and *** *p* < 0.001).

Changes in oxidative stress were assessed via hydrogen peroxide levels (Figure 7). Non-diabetic fibroblasts cultured on diabetic collagen with and without EPAC treatment exhibited significantly less hydrogen peroxide compared to non-diabetic cells cultured on non-diabetic collagen (Figure 7A; one-way ANOVA *p* = 0.0438). Similarly, diabetic fibroblasts on diabetic collagen with or without EPAC treatment displayed significantly less hydrogen peroxide compared to cells on non-diabetic collagen (Figure 7B; one-way ANOVA *p* = 0.0014). In contrast, non-diabetic and diabetic RKO fibroblasts did not exhibit any changes in hydrogen peroxide between the different treatment groups (Supplementary Figure S5; one-way ANOVA *p* = 0.9934 and *p* = 0.9093, respectively). Hydrogen peroxide levels did not change between the different treatment groups in Rap1a KO fibroblasts (Figure 7C; one-way ANOVA *p* = 0.9748). The data indicated that cells with RAGE and Rap1a displayed a decrease in hydrogen peroxide levels with exposure to increased amounts of AGEs embedded within collagen.

## 3. Discussion

Prior work demonstrated RAGE signaling activated by exogenous AGEs can be altered by Rap1a activity [33]. This study did not account for the influence of endogenous collagenous AGEs present in significantly higher levels in the diabetic ECM [20]. Extracted diabetic collagen was used to determined (1) diabetic collagen induced more AGE/RAGE signaling in cardiac fibroblasts than non-diabetic collagen and (2) Rap1a contributed to the changes induced by endogenous AGEs within diabetic collagen. 

Diabetic cardiac fibroblasts had greater RAGE-mediated responses to endogenous AGEs bound to isolated collagen. Significantly higher expression of RAGE and RAGE signaling proteins, p-PKC-ζ and p-ERK1/2, were noted in diabetic cells com-pared to non-diabetic cells. Furthermore, expression of downstream RAGE associated proteins, α-SMA and SOD-2, was significantly greater in diabetic cells than non-diabetic and Rap1a KO fibroblasts. Higher levels of p-PKC-ζ and p-ERK1/2 in di-abetic cells could be due to higher basal levels of RAGE expression possibly contrib-uting to greater expression of α-SMA and SOD-2 in diabetic fibroblasts [33]. Lin et al., 2006 showed AGE treated mesangial cells had increased expression of p-ERK and fi-bronectin, and changes in expression were prevented with SOD pretreatment [12]. Lack of differences between non-diabetic RKO and diabetic RKO fibroblasts supported that diabetic cardiac fibroblasts could be “primed” to respond to elevated RAGE signaling as a result of increased AGE presence. 

A positive correlation existed between high endogenous AGE levels in diabetic collagen and increased RAGE signaling proteins. Changes were further elevated with increased EPAC-mediated Rap1a activity. Cardiac fibroblasts cultured on diabetic collagen displayed higher levels of RAGE signaling proteins (Rap1a, RAGE, and PKC-ζ) compared to fibroblasts on non-diabetic collagen. These results were likely caused by higher levels of endogenous AGES in diabetic collagen activating RAGE signaling as well as contributing to a positive feedback mechanism [20,45,46]. Supporting evidence was noted in the lack of detectable Rap1a and changes in p-PKC-ζ expression in RKO fibroblasts. In addition, EPAC treatment increased Rap1a and RAGE signaling proteins in Rap1a wildtype cardiac fibroblasts while no changes occurred in Rap1a KO cells. Correlating prior data showed increased Rap1a activity crosses the RAGE cascade to increase expression of RAGE signaling proteins [33]. Overall, the results indicated the presence of collagen with endogenous AGEs modulated increased RAGE signaling al-lowing for Rap1a to overlap and amplify the signaling cascade [33]. Changes in up-stream RAGE associated signaling proteins were demonstrated to impact downstream RAGE signaling protein expression. 

Endogenous AGEs within the diabetic ECM increased AGE/RAGE-mediated α-SMA expression, a myofibroblast. α-SMA expression analysis revealed significantly higher expression in non-diabetic fibroblasts cultured on diabetic collagen. Similar trends were noted in AGE/RAGE-mediated increases in α-SMA expression linked to fibrosis [1,14,47,48]. α-SMA expression was further increased with EPAC treatment in non-diabetic cells. Conversely, significant increases in α-SMA expression were not ob-served in Rap1a KO fibroblasts, suggesting Rap1a may be connected to AGE/RAGE myofibroblast transition [5,47,49]. Furthermore, increased α-SMA expression appeared reliant on the presence of the diabetic ECM. A previous study showed cardiac fibro-blasts cultured on plastic had decreased α-SMA expression with exogenous AGE treatment [33]. Thus, further supporting cardiac fibroblasts and ECM interactions are capable of impacting cellular responses generated by AGE/RAGE signaling [4,49,50].

In contrast, diabetic fibroblasts responded differently when cultured on diabetic collagen. This combination exhibited decreased α-SMA expression, which aligned with previously published data showing a similar trend with exogenous AGE treat-ment [1,33,48]. Elevated RAGE expression in diabetic cells may contribute to a “prim-ing” mechanism leading to sustained RAGE signaling to elicit different temporal- or concentration-dependent responses. Diabetic cells may also exhibit an initial increase in α-SMA within early/acute exposure to diabetic collagen. Overtime with chronically, elevated AGE presence, RAGE signaling may cause diabetic fibroblasts to transition into another phase of response, such as sustained inflammation or oxidative stress. Studies have shown similar results, where α-SMA protein expression can display dif-ferential responses based upon a cell’s physiological parameters, like animal age or pathology, as well as exposure time to exogenous factors like AGEs [1,3,47,48]. Addi-tional studies will need to be conducted to determine if differential responses exhibited by diabetic cells was due to either a chronological or concentration-dependent re-sponse to endogenous AGE exposure.

Exogenous AGEs coupled with diabetic collagen induced an increase in p-NF-κB expression which has been linked to inflammation and oxidative stress. Our results in-dicated non-diabetic and diabetic RAGE-positive cells exposed to diabetic collagen had increased p-NF-κB expression, which was further exacerbated with EPAC treatment. Thus, suggesting endogenous AGEs in diabetic collagen can elevate RAGE signaling to promote expression of p-NF-κB-associated inflammation and oxidative stress [28,29,51]. Elevated AGE/RAGE-mediated increases in NF-κB activity also result in elevated tumor necrosis factor-alpha (TNF-α) expression [17,28]. TNF-α, which demonstrated to generate increased free radical (O2-) production and further promote NF-κB activity, can contribute to a positive feedback loop upregulating RAGE as well as additional TNF-α expression [17,29]. Further investigations are needed to determine changes in TNF-α expression; however, presented studies highlighted a pathway used by RAGE signaling to continually increase to chronic inflammation. Additionally, in-creased p-NF-κB has also been linked to oxidative stress [17,28]. To understand the impact of increased p-NF-κB expression within cardiac fibroblasts, an assessment of oxidative stress was performed.

Expression of SOD-2 and SOD-1, markers of possible oxidative stress, were de-creased with elevated AGE/RAGE signaling. Higher levels of endogenous AGEs in di-abetic collagen caused a decrease in SOD-2 expression, which was further decreased with increased Rap1a activity. Changes in hydrogen peroxide concentration correlated with the observed patterns of SODs expression. Our results suggested decreased oxida-tive stress occurred when AGE/RAGE signaling was increased. However, activation of RAGE has been demonstrated in angiogenic progenitor cells to inhibit SOD-2 expres-sion via microRNA-21 (miR-21) [52]. Additional studies have shown AGE treatment decreased miR-205 resulting in lowered SOD-2 expression while concomitantly in-creasing ROS production [17,52,53,54]. SODs are key regulators in transforming ROS into hydrogen peroxide, and inhibiting expression of SODs within a cell will reduce the conversion of ROS into hydrogen peroxide to prevent neutralization of oxidative stressors [17,54]. Based on published data, it is likely increased RAGE signaling trig-gered by endogenous AGEs in diabetic collagen increased oxidative stress by downreg-ulating the expression of SODs. Furthermore, significant changes in SOD-2 expression and not SOD-1 expression may indicate more of a mitochondrial mediated oxidative stress than cytoplasmic. More studies are required to determine the mechanism of RAGE-mediated SOD regulation in our model. Additionally, ECM presence was nec-essary for RAGE-mediated changes in SOD expression due to previous studies indi-cating exogenous AGEs did not impact SOD expression when cardiac fibroblasts were cultured on a plastic dish [33]. Overall, the data provided evidence for a more p-NF-κB-directed oxidative stress response and less of a downstream AGE/RAGE-mediated inflammatory response. Additional studies will need to be con-ducted to further understand this mechanism.

Previous studies by our laboratory found RAGE associated proteins, α-SMA and p-PKC-ζ, exhibited a differential response when treated exclusively with exogenous AGEs [33]. Exogenous AGE exposure decreased α-SMA expression, while p-PKC-ζ ex-pression was not significantly changed [33]. We suspected the responses were due, in part, to collagen presence and the endogenous AGEs within the diabetic collagen. ECM components can bind and interact with cells to effect changes in signaling cascades and cellular function [44,55]. For example, vascular endothelial cells binding to colla-gen via α1β1 and α2β1 integrins had altered cAMP and PKC activity leading to in-creased production of actin stress fibers [44]. Similarly, α2β1 integrins on dermal fibro-blasts can interact with collagens causing increased α-SMA expression to regulate fi-broblast contractility [56,57]. Based on this information, we believe the presence of iso-lated collagen could be a contributing factor for the differential responses noted in cardiac fibroblast exposed to endogenous AGEs.

In summary, high levels of endogenous AGEs in combination with diabetic colla-gen induced increased expression of AGE/RAGE signaling proteins to impact cellular events linked to myofibroblast transition and oxidative stress. Furthermore, Rap1a was shown to play a key role in mediating the effect of AGE/RAGE signaling on down-stream protein expression by further exacerbating observed changes in protein expres-sion. This study provided insight into the influence of endogenous AGEs and the dia-betic matrix on cardiac fibroblast protein expression, and the possible implications these changes may have on increasing the risk of heart disease in diabetic individuals.

## 4. Methods

### 4.1. Animal Models

This study utilized male 16-weeks old Leprdb (db/db model; C57BL/6 background) type 2 diabetes mellitus mice (BKS Cg-DOCK7m +/+ Leprdb/J, Jackson Labs; JAX# 00642). These mice were generated by a point mutation in the leptin receptor producing a nonfunctional leptin receptor (resulting in the db/db mouse model). This mutation caused obesity and insulin resistance, which led to the hyperglycemia by 8-weeks of age and overt diabetes by 12-weeks of age. Heterozygous male mice (referred to as non-diabetic) were used as lean controls.

In addition, homozygous RAGE knockout (RKO; C57BL/6 background) 16-week old male were used in this study. Generation of RKO mouse model was done by flanking exons 2–7 with two loxP sites in the same orientation. Exposure to Cre recombinase, via breeding with Cre delete mice, led to the loxP sites and exons 2–7 to be deleted which caused a constitutive, global loss of RAGE mRNA expression [58,59,60]. Global loss of RAGE mRNA resulted in a loss of RAGE signaling within the mouse model. In addition, a reversely orientated transcriptional EGFP reporter gene was inserted into intron 7. An EGFP PCR genotyping reaction was conducted to act as a positive control for RAGE knockout mice. Documentation of loss of genomic RAGE and expression of EGFP was presented in Burr et al., 2020 (https://doi.org/10.6084/m9.figshare.11299253) [4]. Diabetic and non-diabetic RKO mice were generated by crossbreeding RKO mice to heterozygous (non-diabetic) mice [4,58,59].

16-week old Rap1a knockout male mice (Rap1a KO; C57BL/6 background) were used for this study. Rap1a mouse model was created by inserting a neomycin resistant gene downstream of exon 4 of RAP1A in the opposite (3′-5′) orientation. The resistance gene was inserted by introducing a targeting vector (a 0.95 kb Pyull-Ndel fragment) that caused a disruption of Rap1a mRNA expression [61]. Only non-diabetic Rap1a KO mice were used in this study due to the inability to successfully cross db/db mice with Rap1a KO mice to produce diabetic Rap1a KO mice.

### 4.2. Animal Care

Mice were housed under standard environmental conditions on commercial mouse chow and tap water ad libitum with a 12 h/12 h light/dark cycle. The studies conducted followed the principles of the National Institutes of Health “Guide for the Care and Use of Laboratory Animals,” (NIH publication No. 85–12, revised 1996). The University of Mississippi Institutional Animal Care and Use Committee approved the animal protocol used for this study (protocol #20–017). Euthanasia consisted of anesthesia by CO_2_ followed by cervical dislocation as a secondary method of euthanasia. After euthanasia, body weight and non-fasting blood glucose levels were taken, and then the chest cavity was opened, and the heart was quickly removed, weighed, and used for future experimental studies (Table 1). N-values for all studies were indicated in corresponding figure legends.

### 4.3. Cardiac Fibroblast Isolation

Freshly excised ventricles with the atriums removed were cut into approximately 5 mm sections and placed in a collagenase-trypsin enzymatic solution (0.1% Trypsin, Gibco; 100 U/mL collagenase II, Worthington Biochemical) under sterile conditions^2^. Cardiac tissue was continually mixed until the tissue became a single cell suspension. The cell suspension was resuspended in high glucose DMEM media (high glucose media; Dulbecco’s Modified Eagles Medium (DMEM) containing 4.5 g/L glucose, L-glutamine, sodium pyruvate, and supplemented with 14.9 mM HEPES, 14.2 mM NaHCO3, 1% L-glutamine, 0.02% Primocin™ (Thermo Fisher), and 15% heat-inactivated fetal bovine serum (FBS)) and incubated in 5% CO_2_ at 37 °C. After 24 h, non-adherent cells were washed away, and attached cells were cultured in the appropriate media (non-diabetic and Rap1a KO fibroblasts: low glucose media (1 g glucose/L) and diabetic fibroblasts: high glucose (4.5 g glucose/L)). For simplicity, fibroblasts isolated from a specific mouse model are referred to as that name; for example, fibroblasts isolated from diabetic mice are referred to as diabetic cardiac fibroblasts. Cardiac fibroblast isolations consisted, on average, of using two-three hearts per isolation, which was considered as one sample (*n* = 1). Isolation n-values are indicated in figure legends of corresponding figures. To note, fibroblasts isolated from specific mouse models did not show any differences in morphology or culturing of fibroblasts; images depicting cardiac fibroblasts from specific mouse models can be found in data repository (10.6084/m9.figshare.13369535).

### 4.4. Cell Culture and Experimental Treatment

Freshly isolated or passage 0 (P0) cardiac fibroblasts were cultured until 90–95% confluency and passaged to P1 using 0.25% trypsin and 0.1% ethylenediaminetetraacetic acid (trypsin/EDTA) solution (Life Technology). Fibroblasts were plated onto collagen coated 60 mm cell culture dishes. Dishes were coated with collagen isolated from either non-diabetic or diabetic mouse tails. For collagen coating of plates, 100 µL of collagen of either non-diabetic or diabetic collagen was evenly spread, via a sterile cell scraper, across a 60 mm dish and incubated at 37 °C for 1 h. Excess collagen was removed by washing collagen plates with sterile 1X PBS, twice. P1 fibroblasts were cultured until confluency, washed with sterile 1X PBS, and incubated in starving DMEM (0.01% FBS) for 24 h. After 24 h, starving media was replaced 1 h before the addition of EPAC (8-(4-Chlorophenylthio)-2′-O-methyladenosine 3′,5′-cyclic monophosphate monosodium hydrate; 100 µM, Sigma Aldrich). EPAC was added to fibroblasts and incubated for 24 h, followed by isolation of proteins. P1 cardiac fibroblasts were used to ensure fibroblasts maintained an in vivo phenotype within the in vitro system [62,63,64]. A schematic of the experimental design can be located in data repository (10.6084/m9.figshare.13369535). 

### 4.5. Collagen Extraction

Tails from 16-week-old non-diabetic and diabetic mice were used to isolate collagen [65]. Tails were collected at the time of euthanasia and stored at −20 °C until use. The four major tail tendons were removed and placed in 150 mL acetic acid (1:1000 dilution in dH_2_O). The tendons were cut into smaller (~5 mm) pieces, and the tendon solution was mixed for 3 days at 4 °C. Hereinafter, all steps were conducted using sterile conditions. The tendon solution was centrifuged at 3000× *g* for 30 min at 4 °C. The supernatant was transferred to new 50 mL conical tubes and spun again under the same conditions. Collagen supernatant was removed and stored at 4 °C. Sircol^TM^ Soluble Collagen Assay Kit (Biocolor Ltd.) was used following the manufacturer’s directions to determine collagen concentration. The concentration of collagen used to coat the plates in this study was 100 µg/mL.

### 4.6. Protein Isolation and Western Blot Analysis

Modified Hunter’s buffer (MHB; 0.5 mM orthovanadate, 75 mM NaCl, 5 mM tris pH 7.4, 0.5 mM EDTA, 0.5 mM EGTA, 1% Triton X-100, 0.25% NP-40, and freshly added Halt Protease Inhibitor Cocktail (100X; Thermo Fisher)) was used to isolate total protein from cardiac fibroblasts. Fibroblasts and MHB were incubated on ice for 10 min, followed by probe-sonication. Cell lysates were centrifuged for 15 min at 32,000× *g* at 4 °C and the supernatant was stored at −80 °C. A bicinchoninic acid assay (BCA; Pierce Biotechnology) was used according to the manufacturer’s instructions to determine protein concentration. SDS-page gels were loaded with 10 µg of protein per sample for Western blot analysis. Primary antibodies used were as followed: monoclonal α-smooth muscle action (α-SMA, 42 kDa; 1:400; Sigma Aldrich 2547), phosphorylated nuclear factor kappa-light-chain-enhancer of activated B cells (p-NF-κB, 65 kDa; Ser 536; 1:400; Santa Cruz Biotechnology sc-136548), superoxide dismutase 2 (SOD-2, 25 kDa;1:400; Santa Cruz Biotechnology sc-133134), superoxide dismutase 1 (SOD-1, 23 kDa;1:400; Santa Cruz Biotechnology sc-17767), repressor activator protein 1a (Rap1a, 21 kDa; 1:400; abcam ab96223), phosphorylated extracellular signal-regulated kinase (p-ERK1/2, 42 and 44 kDa; Tyr 204; 1:400; Santa Cruz Biotechnology sc-7383), extracellular signal-regulated kinase 1 and 2 (ERK1/2, 42 and 44 kDa; 1:400; Santa Cruz Biotechnology sc-271269 and sc-1647), phosphorylated protein kinase C zeta (PKC-ζ, 72 kDa; phospho T560; 1:400; Abcam ab62372), and beta-tubulin (β-tubulin, 55kDa; 1:400; Santa Cruz Biotechnology sc-398937). Total protein was labeled with Brilliant Blue Coomassie stain. β-tubulin was used as a loading control to normalize protein expression for α-SMA, p-NF-κB, SOD-2, Rap1a, and p-PKC-ζ. Total ERK1/2 was utilized to normalize p-ERK1/2 protein expression. In order to assess for expression of all desired proteins, membranes were stripped and reprobed in order to maximize the use of our limited sample volume as well as reduce the total number of mice needed for this experiment. The iBRIGHT imaging system was used to visualize the Western blots, and Image J was used for analysis.

Representative Western blot images are presented with each figure. Due to logistical constraints, it was decided to run samples of the same genotype on the same membrane in order to assess the impact of the treatment groups on protein expression; therefore, graphs comparing different genotypes (Figure 1 and Figure 2) have representative Western blot images presented as non-continuous blots, while graphs comparing different treatments (Figure 3, Figure 4, Figure 5 and Figure 6 and Supplementary Figures S1–S3) have blot images presented as a continuous blot. Due to the number of proteins examined and the total volume of protein samples, it was not feasible to run the samples multiple times in order to present representative Western blot images as continuous for all samples. Original Western blot images can be found at 10.6084/m9.figshare.13369535.

### 4.7. Hydrogen Peroxide Assay

The concentration of hydrogen peroxide was determined using protein lysates. The OxiSelect hydrogen peroxide/peroxidase assay kit (BioLabs, STA-344) is an assay that uses a colorimetric probe that reacts with hydrogen peroxide to produce a pink-colored product. This kit was used per the manufacturer’s directions. Protein lysates were diluted 1:2 in assay buffer solution and added to a 96 well plate. A total of 50 μL of hydrogen peroxide working solution was added to each sample and the plate was incubated in the dark for 30 min at room temperature. A spectrometer was used to assess the colorimetric assay using a wavelength of 540 nm. The concentration of hydrogen peroxide was determined using a standard curve.

### 4.8. Statistical Analysis

Statistical analysis was conducted using Graph Prism software, version 9.0.2. Protein expression was normalized to β-tubulin (α-SMA, p-NF-κB, SOD-2, Rap1a, and p-PKC-ζ) or total ERK1/2 (p-ERK1/2) before statistical analysis was conducted. A Student’s *t*-test was utilized to determine the significance of the data presented in Figure 2. One-way ANOVA followed by a Fisher’s Protected Least Significant Difference post hoc were conducted with data presented in Figure 1, Figure 3, Figure 4, Figure 5, Figure 6 and Figure 7 and Supplementary Figures S1–S3 to determine significant differences. The group size is not the same across the different treatment groups due to a couple of reasons: (1) the number of cell isolations varied based on mice availability, not all genotypes were generated in the same birth ratio; (2) not all cell isolations grew to confluency at P1 and were thus were not used in the experiments; (3) since each treatment group consisted of one 60 mm dish there could be variations in cell density, health, or other uncontrolled factors impacting the cells which could have led that treatment group not being considered during the analysis of the data. While varied group sizes could ultimately impact statistical analysis, we feel that the overall sample size was high enough to mitigate any impact on statistical analysis.

## Figures and Tables

**Table 1:** Physiology measurements of mice used in this study. Average values for ratio of heart weight/body weight and blood glucose for mice. On average, 2–3 mouse hearts were used for one cardiac fibroblast isolation. Statistical analysis consisted of one-way ANOVA followed by a Dunnett’s post hoc compared to non-diabetic mice (**** *p* < 0.0001).

	Heart Weight (g)	Blood Glucose (mg/dL)
	Body Weight (g)	
Non-Diabetic (*n* = 30)	0.0038 ± 5.362 × 10^−5^	198.9 ± 5.187
Diabetic (*n* = 16)	0.0022 ± 7.378 × 10^−5^ ****	511.4 ± 27.98 ****
Non-Diabetic RKO (*n* = 24)	0.0037 ± 7.349 × 10^−5^	193.1 ± 7.148
Diabetic RKO (*n* = 14)	0.0022 ± 3.472 × 10^−5^ ****	428.2 ± 27.99 ****
Rap1a KO (*n* = 27)	0.0040 ± 7.297 × 10^−5^	187.8 ± 7.007

## Data Availability

All relevant data are included within the manuscript. Supplemental and original blot images are available: 10.6084/m9.figshare.13369535.

## Supplementary Materials

The following are available online at https://www.mdpi.com/article/10.3390/ijms23094480/s1.
