# Supplementary material for: Rap1a Activity Elevated the Impact of Endogenous AGEs in Diabetic Collagen to Stimulate Increased Myofibroblast Transition and Oxidative Stress
Source: Int J Mol Sci. 2022 Apr 19;23(9):4480. doi: 10.3390/ijms23094480 (PMC9101126; doi:10.3390/ijms23094480)
Supplement: Supplementary file 1 [file ijms-23-04480-s001.zip › ijms-1655658-supplementary.pdf]

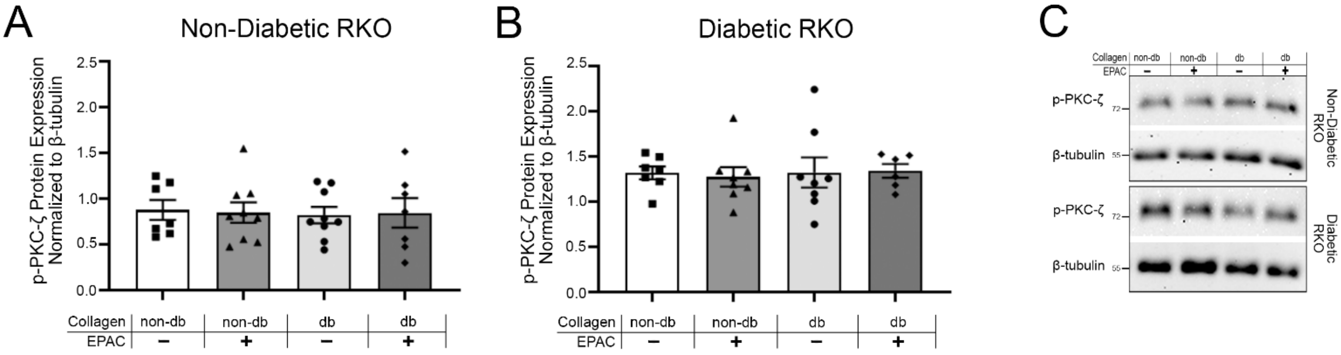

Figure S1. Neither endogenous AGE exposure nor Rap1a activation caused a shift in p-PKC-ζ protein expression in RKO fibroblasts. Non-diabetic and diabetic RKO cardiac fibroblasts were isolated and cultured either on non-diabetic or diabetic collagen. Fibroblasts were either untreated or treated with EPAC (100 μM). Protein was collected from fibroblasts and used to determine changes in (**A**,**B**) p-PKC-ζ (72 kDa) protein expression. (**C**) Representative Western blot images for p-PKC-ζ in RKO fibroblasts. Protein expression was normalized to β-tubulin (55 kDa) and mean ± SEM are depicted on graph (n = 7–8). Statistical analysis consisted of one-way ANOVA followed by a Fisher's Protected Least Significant Difference post hoc.

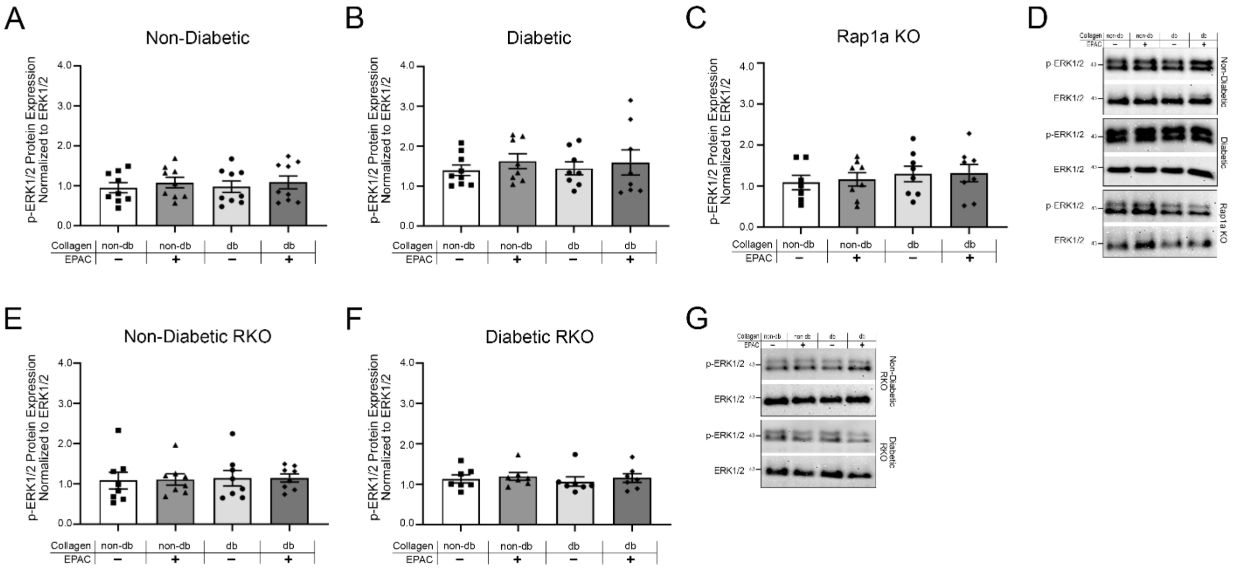

Figure S2. Exposure to endogenous AGEs with or without EPAC did not induce significant changes in p-ERK1/2 protein expression. Cardiac fibroblasts were isolated from (**A**) non-diabetic, (**B**) diabetic, (**C**) Rap1a KO, (**E**) non-diabetic RKO, and (**F**) diabetic RKO mouse hearts and were cultured on either non-diabetic or diabetic collagen. Cells were treated with EPAC and used to isolate protein to assess for changes in p-ERK1/2 (44 and 42 kDa, respectively) protein expression. Expression of p-ERK1/2 was normalized to total ERK1/2 and mean ± SEM were displayed in graphs (n = 7–11). (**D**,**G**) Representative Western blot images for p-ERK1/2 and total ERK1/2 in cardiac fibroblasts. Statistical analysis consisted of a one-way ANOVA followed by a Fisher's Protected Least Significant Difference post hoc.

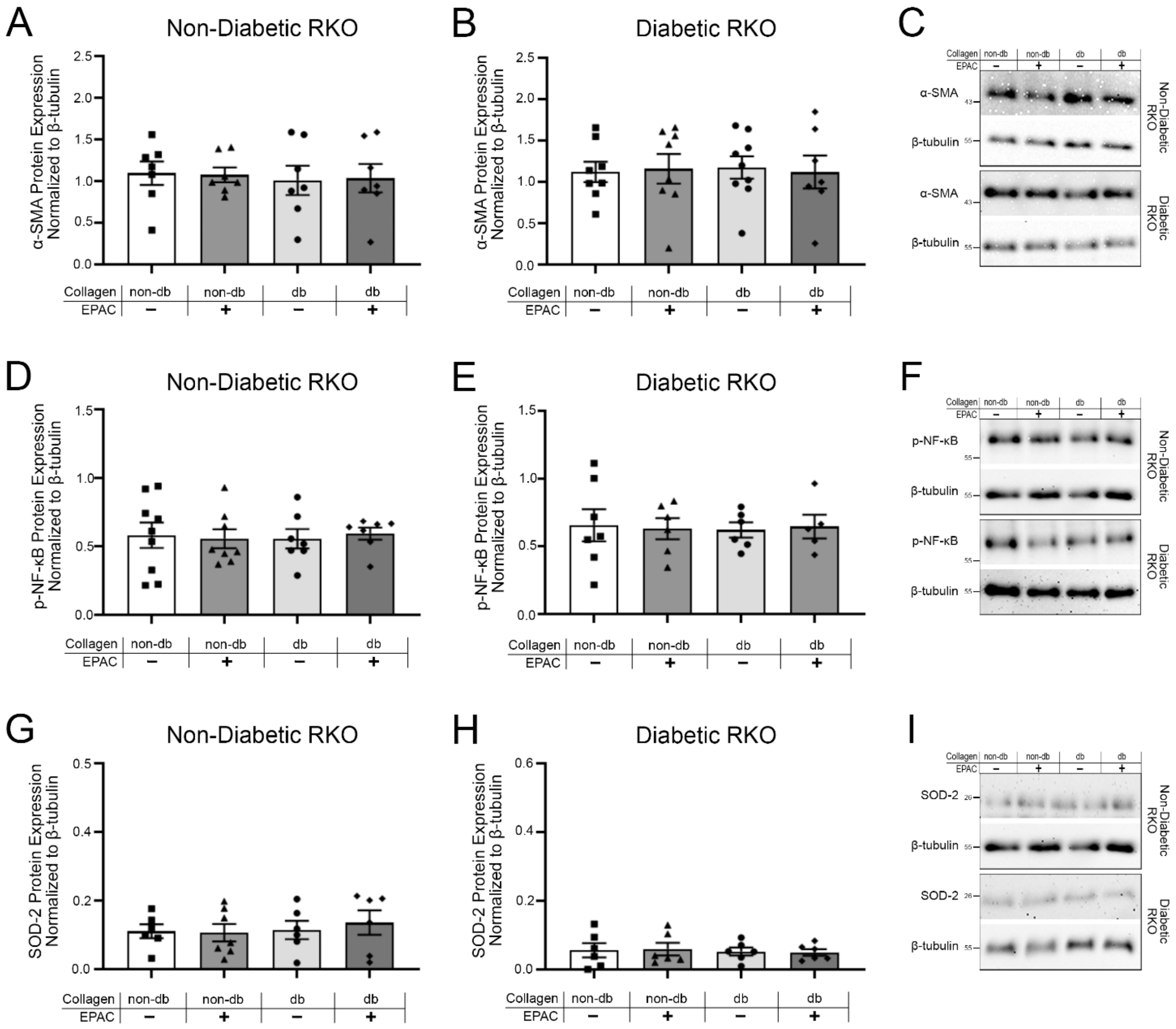

Figure S3. RKO cardiac fibroblasts did not display changes in expression of AGE/RAGE-associated proteins when exposed to endogenous AGEs or increased Rap1a activity. Non-diabetic RKO and diabetic RKO cardiac fibroblasts were isolated and P1 fibroblasts were cultured on either non-diabetic or diabetic collagen. Fibroblasts were either left untreated or treated with EPAC (100 μM) and used to isolate total protein. Changes in protein expression for (**A**,**B**) α-SMA (42 kDa), (**D**,**E**) p-NF-κB (65 kDa), and (**G**,**H**) SOD-2 (25 kDa) were determined for both (**A**,**D**,**G**) non-diabetic and (**B**,**E**,**H**) diabetic RKO fibroblasts. Protein expression was normalized to β-tubulin (55 kDa). Values within graphs are mean ± SEM (n = 6–9) and one-way ANOVA followed by a Fisher's Protected Least Significant Difference post hoc was utilized to determine significance. (**C**,**F**,**I**) Representative Western blot images are depicted for α-SMA, p-NF-κB, and SOD-2 expression.

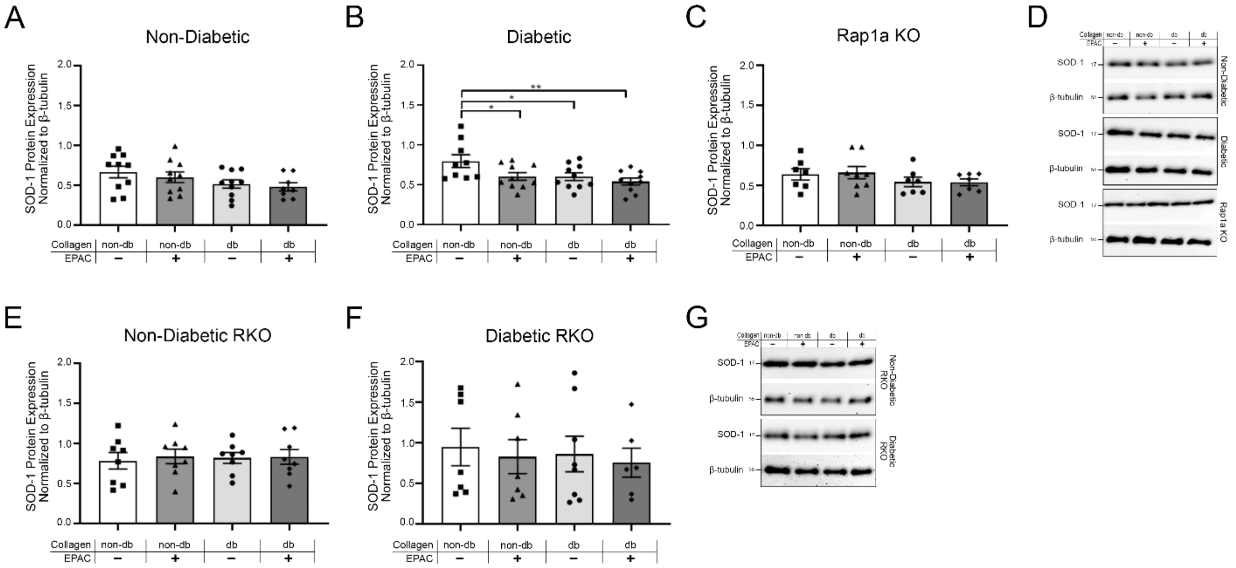

Figure S4. Diabetic collagen caused a slightly decreased SOD-1 expression in RAGE wildtype cardiac fibroblasts. Isolated cardiac fibroblasts from (**A**) non-diabetic, (**B**) diabetic, (**C**) Rap1a KO, (**E**) non-diabetic RKO, and (**F**) diabetic RKO were cultured on non-diabetic or diabetic collagen and treated with EPAC (100 μM). Total protein was collected, and expression of oxidative maker SOD-1 (23 kDa) was determined. SOD-1 protein expression was normalized to β-tubulin (55 kDa) and mean + SEM are presented within the graphs (n = 5–10). One-way ANOVA followed by a Fisher's Least Significant Difference post hoc was used to determine significant differences ($p < 0.05$ and * $p < 0.01$). (**D** and **G**) Representative Western blot images are presented for SOD-1 expression.

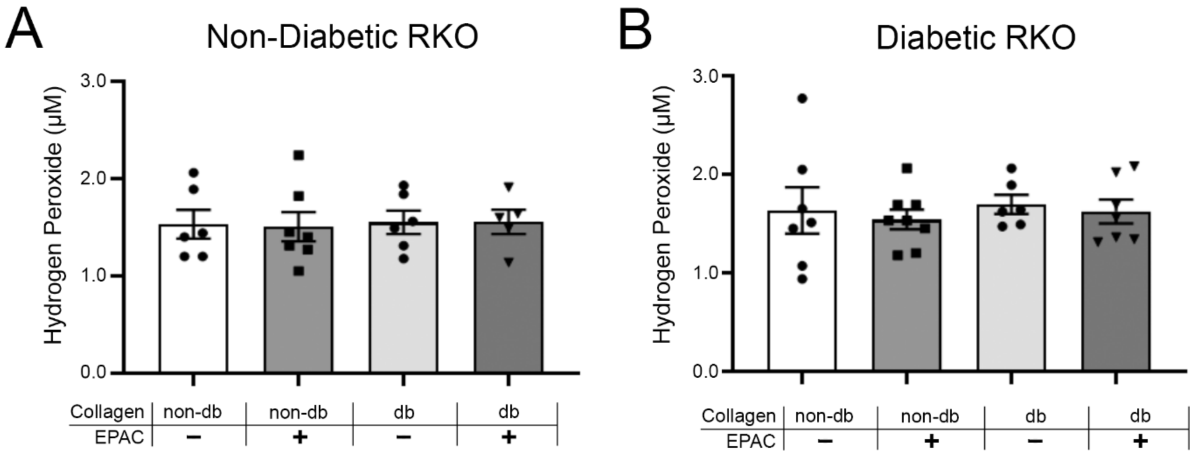

Figure S5. Exposure to endogenous AGEs and EPAC did not induce changes in hydrogen peroxide concentration in non-diabetic and diabetic RKO cardiac fibroblasts. (**A**) Non-diabetic RKO and (**B**) diabetic cardiac fibroblasts (P!) were cultured on either non-diabetic or diabetic collagen and treated with EPAC (100 μM). Protein lysates were assessed for hydrogen peroxide concentration (μM) and mean ± SEM were depicted on graphs (n = 5–8). Statistical analysis consisted of a one-way ANOVA followed by a Fisher's Protected Least Significant Difference post hoc.
